# Ischaemic mitral regurgitation: The effects of ring annuloplasty and suture annuloplasty repair techniques on left ventricular re-remodeling

**DOI:** 10.12669/pjms.291.2760

**Published:** 2013

**Authors:** Cemalettin Aydin, Ibrahim Kara, Yasin Ay, Bekir Inan, Halil Basel, Mehmet Yanartas, Rahmi Zeybek

**Affiliations:** 1 Cemalettin Aydin, Department of Cardiovascular Surgery, Bezmialem Vakif University, Fatih, Istanbul, Turkey.; 2 Ibrahim Kara, Private Emsey Hospital, Cardiovascular Surgery Clinic, Pendik, Istanbul, Turkey.; 3 Yasin Ay, Department of Cardiovascular Surgery, Bezmialem Vakif University, Fatih, Istanbul, Turkey.; 4 Bekir Inan, Department of Cardiovascular Surgery, Bezmialem Vakif University, Fatih, Istanbul, Turkey.; 5 Halil Basel, Department of Cardiovascular Surgery, Bezmialem Vakif University, Fatih, Istanbul, Turkey.; 6 Mehmet Yanartas, Kartal Koşuyolu Research and Training Hospital, Cardiovascular Surgery Clinic, Istanbul, Turkey.; 7 Rahmi Zeybek, Department of Cardiovascular Surgery, Bezmialem Vakif University, Fatih, Istanbul, Turkey.

**Keywords:** Ischaemic mitral regurgitation, Coronary artery bypass graft, Mitral annuloplasty

## Abstract

***Objective: ***To examine the mid-term results of patients on whom a coronary revascularization as well as a mitral ring and suture annuloplasty have been performed due to coronary artery disease (CAD) and ischaemic mitral regurgitation (IMR).

***Methodology:*** Totally 73 patients on whom a revascularization and a mitral valve repair due to CAD and IMR had been performed in our clinic between 2000-2008 were included in the study. Patients were divided into two groups one of which included 38 patients (52.05%) on whom a coronary artery bypass graft (CABG) and a ring annuloplasty on the mitral valve had been performed (Group 1) and the other one 35 patients (47.95%) on whom only suture annuloplasty as well as a CABG had been performed (Group 2). The study was planned retrospectively and study data have been obtained by screening the hospital registries retrospectively. In the mid-term, patients were invited for a check and their intragroup and intergroup echocardiographic parameters and functional capacities were assessed statistically.

***Results:*** In pre-operational and post-operational intragroup assessment in terms of echocardiographic findings; although LVEDD, LVESD, EDV, PAP and the degree of recurrent MR have been decreased in both groups, the decrease in LVESD and PAP and the low degree of recurrent MR were statistically significant in Group 1 patients (p=0.047, p=0.023, p=0.01, respectively). When the mid-term intergroup echocardiograpic findings were assessed; PAP and recurrent MR have been determined statistically lower in Group 1 patients (p=0.005, p=0.08, respectively). The length of intensive care unit stay, length of hospitalization and length of detachment from respiratory support were statistically significantly longer in ring annuloplasty performed group (p=0.012, p=0.033, p=0.029, respectively).

***Conclusions:*** In moderate to severe IMR patients, a positive contribution can be provided to ventricular remodeling by a ring annuloplasty through a significant decrease in left ventricular diameter and a low recurrent MR and PAP.

## Introduction

 Mitral valve regurgitation is a serious health problem which is observed in 80% of the population in mild to severe forms and may develop due to numerous causes. Ischaemic mitral regurgitation (IMR) is a complication of coronary artery disease. It’s not a valvular but a ventricular disease and develops along with a regional and global left ventricular remodeling that occurs after an Acute Myocardial Infarction (AMI).^1^^-^^3^ In mitral regurgitation, there are various surgical techniques. Although the repair techniques includes ring annuloplasty, there are several other repair techniques in which a ring device is not implemented.

 We aimed to assess the mid-term results of the two goups, in which the mitral repair in company with a coronary bypass surgery had been performed either by using a ring device or not in patients with mitral regurgitation in company with a coronary artery disease, retrospectively.

## Methodology

 Totally 73 patients (48 male, 25 women, mean age 61.78±8.44, range 45-78 years) who were operated due to a coronary artery disease and IMR between 2000-2008 and on whom a combined CABG and a mitral valve repair had been performed were included in the study. The surgical procedures performed on the patients were assessed retrospectively and the patients were allocated into two groups which were CABG and the repair of mitral valve by using a ring device (Group 1, n=38) and CABG and the repair of mitral valve without using a ring device (Group 2, n=35). In Group 1, 27 of the patients were male (71.1%) and 11 were women (28.9%). In Group 2, 21 of the patients were male (60.0%) and 13 were women (40.0%). Pre-operational characteristics of the groups are given in Table-I. Written informed consent form was obtained from all the patients included in the study. The study was approved by Ethics Committee of the Bezmialem Vakif University.

**Table-I:** Evaluation of groups for preoperative characteristics

*Variables*	*Group 1 (n=38)*	*Gorup 2 (n=35)*	*p*
Age, mean±SD, year	63.02±8.41	60.43±8.33	0.191
Gender, n (%)			
Male Female	27 (71.1) 11 (28.9)	21 (60.0) 14 (40.0)	0.320
Euroscore (st), mean±SD	7.85±2.88	8.98±4.39	0.195
Euroscore (Lo), mean±SD	11,47±9,05	12,50±9,08	0,628
PHT, mean±SD, mmHg	49.48±20.09	49.55±12.87	0.986
NYHA class, mean±SD, (medyan)	3.60±0.64, (4)	3.48±0.50, (3)	0.176
COPD, n (%)	11 (28.9)	14 (40.0)	0.320
Rhythm, n (%)			
Sinus rhythm AF rhythm	24 (63.2) 14 (36.8)	31 (88.6) 4 (11.4)	0.015*
Diabetes mellitus, n (%)	18 (47.4)	14 (40.0)	0.526
Hypertension, n (%)	29 (76.3)	20 (57.1)	0.081
Previous neurological event, n (%)	2 (5.3)	1 (2.9)	1.000
Critical preoperative state, n (%)	8 (21.1)	7 (20.0)	0.911
Unstable angina, n (%)	16 (42.1)	19 (54.3)	0.298

COPD: Chronic obstructive pulmonary disease, NYHA: New York Heart Association,PHT: Pulmonary hypertension, mean±SD: mean± standard deviation

 General characteristics of the patients (age, gender), Euroscore, pre-operative risk factors of NYHA (COPD, diabetes, hypertension, neurological dysfunction, unstable angina, new MI, left ventricle dysfunctions, pulmonary hypertension), pre-operative and post-operative rhythm and echocardiographic parameters (LVEDD, LVESD, EDV, ESV, LA, PAP, EF, recurrent MR), operational findings (heat, cct, tpt, antegrade and retrograde cardioplagia), post-operative complications (extubation period neurological case, a respiratory case, renal disorders, rhythm, IABP, revision, operative mortality), length of intensive care unit stay and length of hospitalization were recorded. The average duration for follow-up in patients with a mitral repair with or without using a ring device were; 22.6±20.86 (2-97) months and 11.53±19.48 (0.5-78), respectively. These periods were identified as the mid-term for groups and the patients were called for a control. A mid-term echocardiography were performed and both intragroup’s pre- and post-operational and intergroup’s post-operational echocardiographic data were assessed. 

 The mitral valve of the patients with echographically pre-operative moderate to severe IMR were intervened. The repair technique for mitral valve and the use of ring device were decided in accordance with the assessment of mitral valve structure by the surgeon during the operation. Mitral regurgitation were graded in echocardiographic method as; 0: no regurgitation, 1°: mild regurgitation, 2°: mild to moderate regurgitation, 3°: moderate regurgitation and 4°: severe regurgitation.

 The repair techniques and ring types used in both groups are shown in Table-II. In post-operative period, all the patients were monitored by an invasive arterial monitor and patients in need of an inotrop and intra-aortic balloon pump (IABP) were provided. The pulses of patients who were provided an IABP were checked by a digital examination and by doppler, when necessary. Sefazolin sodium vials of 1000 mg have been administered routinely in six hour intervals for prophylaxis. In accordance with the intensity of the invasive intervention, a second antibiotic was added in patients at risk (DM, obesity, osteoporosis etc), when deemed necessary.

***Surgical Technique: ***A median sternotomy was performed on all of the patients under general anesthesia. A left internal mammarian artery (LIMA) or saphanous vein were prepared in accordance with the hemodynamic condition of the patient. An arterial cannulation was performed on patients, who were operated by using an extra-corporeal circulation. Cannulation form both caval veins (bicaval) was performed. A venting cannula was placed from the upper right pulmonary vein. A diastolic arrest was performed with isothermic blood cardioplagia with K^+^ given through aortic root following a cross clamp. During the operation, isothermic blood cardioplegia was administered continuously through coronary sinus route. As the common myocardial protection methods, systemic hypothermia (28-32°C), cross clamp, antegrade-retrograde crystalloid with K^+^ or blood cardioplegia were administered. With the start of the total bypass, coronary distal anastomoses were created first. Then, the mitral valve was intervened. Intervention to mitral valve was performed through left atrial incision. In case the left atrium was small, inter-arterial transseptal approach was used to improve mitral valve visibility. In Group 1, a ruptured corda was found in 4 patients and a ruptured papillary muscle in one patient, in Group 2, a ruptured corda was seen in 5 patients.

***Statistical Analysis*** *: *While evaluating the findings from the study, NCSS 2007&PASS 2008 Statistical Software (Utah, USA) program was used for statistical analyses. While evaluating the study data, Student t test has been used for the comparison of the quantitative data and for the inter-group comparison of parameters with normal distribution, and Mann Whitney U test has been used for the inter-group comparison of parameters without a normal distribution, as well as defining statistical methods (Mean, Standard deviation). Paired sample test has been used for the inter-group comparison of the parameters with a normal distribution, and Wilcoxon marking test has been used for the inter-group comparison of parameters without a normal distribution. And for the comparison of the qualitative data, Chi-Square test and Fisher’s exact test have been used. Result were evaluated with 95% confidence interval and at p<0.05 significance level.

## Results

 The mean age of the patients were 61.78±8.44. The follow-up period of the cases varied between 5 days and 97 months, and the mean follow-up period was 19±22.05 months and median value was 9 months. Pre-operational characteristics and comparison of the groups were given in Table-I. In Group one patients, the number of patients with pre-operative AF rhythm [14 patients (36.8%)] was significantly more than those in Group 2 [4 patients (11.4%)] (p=0.015). There was no statistically significant difference between the groups in terms of other pre-operative characteristics and echocardiographic findings (p>0.05).

 In evaluation of the peri-operative findings, the length of intensive care unit stay, the length of hospitalization and period of weaning from the respiratory support after the operation of the ring annuloplasty group were statistically significantly longer than those of the suture annuloplasty group (p=0.012, p=0.033, p=0.029, respectively). There were no statistically significantly difference between the groups in terms of CCT and TPT (p>0.05).

**Table-II:** Evaluation of groups according to the applied mitral procedure

*Mitral repair procedure*	*Group 1 (n=38)*	*Group 2 (n=35)*
P2 plication + St. Jude ring annuloplasty, n (%)	9 (23.7)	-
Kalangos ring annuloplasty, n (%)	2 (5.26)	-
St Jude ring annuloplasty, n (%)	21 (55.26)	-
Alfieri repair + St Jude ring annuloplasty, n (%)	3 (7.89)	-
Mc Goon annuloplasty + St Jude ring annuloplasty, n (%)	2 (5.26)	-
Jostra ring annuloplasty, n (%)	1 (2.63)	-
Kay annuloplasty, n (%)	-	27 (77.14)
Wooler annuloplasty, n (%)	-	2 (5.71)
Chordoplasty, n (%)	-	1 (2.86)
Artificial chorda and Mc Goon annuloplasty, n (%)	-	1 (2.86)
Quadroangular resection + plication, n (%)	-	3 (8.57)
Alfieri repair, n (%)	-	1 (2.86)

**Table-III:** Assessment of the perioperative characteristics

	*Group I*	*Group II*	*p*
	*mean±SD*	*mean±SD*	
CCT, minute	98.05±29.10	89.29±23.99	0.164
TPT, minute	135.55±28.64	128.91±35.90	0.388
Length of intensive care unit stay, days	±	±	0,012*
Length of hospital stay, day	±	±	0.033*
Extubation time, hour, (Median)	15.02±20.48 (8)	7.21±3.74 (3)	0.029*
Cardioplegia Antegrade, n, (%)	38 (100.0)	34 (97.1)	0.479
Retrograde, n (%)	31 (81.6)	29 (82.9)	0.887

CCT: Cross clamp time, TPT: Total perfusion time

*p<0.05

No statistically significantly difference was determined between the groups in terms of post-operative neurological cases, renal disorders, IABP support, revision and hospital mortality rate (p>0.05) (Table-IV). The rate of respiratory cases was significantly hiher in post-operative Group 1 patients (p=0.025) and we believe that this is related with the seriously longer extubation period of Group 1 patients. Although the number of patients in Group 1 with pre-operative AF rhythm was significantly higher that of those in Group 2 (p=0.015) (Table-I), the number of patients with post-operative recently developing AF rhythm was significantly higher in Group 2 (p=0.009) (Table-IV). In pre-operational and post-operational intragroup assessment in terms of echocardiographic findings; although LVEDD, LVESD, EDV, PAP and the degree of recurrent MR have been decreased in both groups, the decrease in LVESD and PAP and the low degree of recurrent MR were statistically significant in Group 1 patients (p=0.047, p=0.023, p=0.01, respectively). When the mid-term intergroup echocardiograpic findings were assessed; PAP and recurrent MR have been determined statistically lower in Group 1 patients (p=0.005, p=0.008, respectively) (Table-V).

## Discussion

 Ischaemic mitral regurgitation is an important problem in cardiac surgery in terms of treatment plan. The surgical approach for the patients with moderate to serious IMR may be in different ways such as coronary revascularization, mitral valve replacement or revascularization combined with valvuloplasty. In certain series, the operative mortality of the cases with mitral valve repair has been found lower than those of the cases with valve replacement and in addition to that, it’s been demonstrated that the ventricular functions were protected better in cases with valve repair, the valvular complications were observed less and the long-term survival rate was higher.^4^ In another study, it’s been observed that mitral repair was significantly associated with higher early survivals in patients with chronic ischaemic mitral regurgitation due to the left ventricular dysfunction or papillary muscle infarction.^5^

**Table-IV:** Evaluation of the post-operative complications

	*Group I*	*Group II*	*p*
	* n=38*	* n=35*	
Neurological events, n (%)	3 (7.9)	1 (2.9)	0.617
Respiratory event, n (%)	17 (44.7)	7 (20.0)	0.025*
Renal disorder, n (%)	9 (23.7)	6 (17.1)	0.490
Rhythm event, n (%)	25 (65.8)	23 (65.7)	0.995
IABP, n (%)	10 (26.3)	4 (11.4)	0.107
Revision, n (%)	2 (5.3)	1 (2.9)	1.000
Postoperative new-onset AFR, n (%)	0 (0)	6 (17.1)	0.009
Hospital mortality	2 (5.3)	2 (5.7)	1.000

IABP: Intra-aortic balloon pump, AFR: Atrial fibrillation rhythm

* p<0.05

**Table-V:** Comparison of intragroup and intergroup for preoperative and postoperative echocardiographic data

*Variables*	*Group 1 (n=38)* *mean±SD*	*Group 2 (n=35)* *mean±SD*	*p* ^b^
LVESD			
Preoperative Postoperative p^a^	4.35±0.89 4.15±0.82 0.337	4.26±0.70 4.21±0.76 0.828	0.631 0.746
LVEDD			
Preoperative Postoperative p^a^	5.78±0.65 5.53±0.59 0.047*	5.73±0.58 5.49±0.60 0.290	0.729 0.775
EDV			
Preoperative Postoperative p^a^	166.01±49.16 151.20±41.92 0.311	157.22±36.98 142.78±36.95 0.387	0.389 0.365
ESV			
Preoperative Postoperative p^a^	89.41±43.06 91.14±39.23 0.975	88.50±44.03 78.35±34.87 0.690	0.929 0.145
LA			
Preoperative Postoperative p^a^	4.47±0.91 4.50±0.79 0.820	4.46±0.72 4.23±0.62 0.098	0.958 0.107
PAP			
Preoperative Postoperative p^a^	51.05±20.27 43.93±11.17 0.023*	50.48±12.86 53.00±16.38 0.259	0.886 0.008**
EF			
Preoperative Postoperative p^a^	0.44±0.14 0.43±0.12 0.315	0.45±0.12 0.45±0.9 0.380	0.743 0.897
recurrent MR degree, (median)			
Preoperative Postoperative p^a^	3.52±0.55, (4) 1.50±0.76, (1) 0.001**	3.41±0.49, (3) 2.13±1.04, (2) 0.001**	0.369 0.005**


EDV: End-diastolic volume, EF: Ejection fraction, MR: Mitral regurgitation,

PAP: Pulmonary artery pressure, LA: Left atrium, ESV: End-systolic volume,

VEDD: Left ventricular end-diastolic diameter, VESD: Left ventricular end-systolic diameter.

p^a^: Comparison of intra-group preoperative and postoperative echocardiographic data.

p^b^: Comparison of inter-group preoperative and postoperative echocardiographic data.

* p<0.05, ** p<0.01

 In MR secondary to ischaemia, the indications of mitral valve intervention is relative. The assessment of the condition of the left ventricle is important for the prognosis and optimal timing of the operation. One should know that a combined CABG and mitral valve procedures would increase surgical mortality and morbidity. There are numerous determinants for the late period prognosis of the patient with mitral valve regurgitation. These are; concomitant CAD, CHF (congestive heart failure), pre-operative NYHA class III-IV symptoms, left ventricular dysfunction (LVD).^6^

 There is a dispute on the valve replacement during CABG in second or third degree MR. The surgeons should see the positive effects of the combined operation (CABG, MVR/Repair) along with the operative risks, on the long-term prognosis. In our cases, MR was moderate to severe, and urgent or semi-elective operations have been performed on patients with on-going anginal compliants.

 After the myocard infarction, there should be a waiting period of 4-6 weeks for the surgery. If the patient can not be stabilized, if the patient has pulmonary oedema and the patient’s conditions is getting worse, then appropriate surgical procedures should be performed despite the high mortality. These procedures include CABG, in combination with chordal repair, valve repair or valve replacement.

 Czer et al have reported that there was less post-operative MR following a ring annuloplasty when compared to a suture annuloplasty in patients with pure annular dilation.^7^ In this study, recovery and remodeling in both survival and survival without complication has been associated with ring annuloplasty. 30-day mortality rate of the study patients was 7.6%. A recently reported operative motality for mitral repair in ischaemic MR is 10-12%.^6^ In our study, operative mortality was 5.3% in Group 1 (2) and 5.7% in Group 2 (2) and there was no statistically significantly difference (p=1.000). In our study, we’ve observed that there was a significant decrease in MR, PAP, LVESD in Group 1 when the post-operative ECHO findings were assessed. In Group 2, there was a significant change only in MR and no significant change has been observed in other parameters.

 In a study, while performing an annuloplasty alone for the functional ischaemic MR has been found dramatically (30%) relevant with 2^nd^ or higher degree MR insidence during the follow-up,^8^ Boling et al have treated severe MRs with perfect results by using a smaller size annuloplasty ring in the dilated heart.^6^ In a study of ischaemic MR, 355 patient who have received CABG only and CABG and repair have been compared, and it’s been found that there was a more decrease in IMR degree in combined group, a low early mortality in CABG alone group and a low long-term survival of unimproved MR following a CABG.^9^ And in our cases, although the preop MR degree was significantly higher, the post-operative MR was much lower in Group 1 The early and late mortality is higher in NYHA 4 and patients with the last stage ischemic heart disease.

 EF is a weak criterion for the assessment in particular in conditions where ischemia and MR exist together or only in MR. In a study, mitral repair and low EF relation has been reviewed retrospectively, and patients with mitral valve repair has been allocated into two groups, with EF>35% and EF<35%. The peri-operative mortality was 2% in Group 1 and 8% in Group 2 (p<0.03), and 5-year survival was 82% in Group 1 and 54% in Group 2.^10^ In our cases, although MR, PAP, LVESD has significantly improved in Group 1 patients, there was a non-significant decrease in EF values. In Group 2, there was no significant change in EF. One should keep in mind that, after the improvement of mitral regurgitation, EF will decrease more in those patients since the after load will increase.

 In Group 1, only the respiratory case from the post-op complications appears significantly high. We believe that this is associated with the significantly longer extubation period. A surgery can be performed on patients with NYHA Class 3-4 functional state if there are no medical contrendications.^11^ When the pre- and post-operative functional capacities of our patients have been assessed, there is a significant improvement in both groups. Based on our study criteria for pre- and post-operative period, EF has been observed to be elevated in patients intervented for acut ischaemic mitral regurgitation in our clinic, depending on the timing of the operation.

 We believe that the ventricular functions of those patients who tolerated MR following an AMI until the time they’re electively operated undergoes a “remodelling” in order to compensate a mitral regurgitation. While there is no difference between groups in terms of mortality, it’s important to minimize the effects of certain foreseeable comorbid factors (DM, peroperative myocard infarction, renal, neurologic and respiratory complications etc) in terms of surgical success. A rapid improvement of ischaemia due to the improvement of the post-operative functional capacity and, if required, an intervention to mitral regurgitation will improve the patient’s quality of life. At this point, it can be considered that patients will benefit considerably from an operation in case the pre-operative surgical assessment is sufficient and the accurate surgical procedure is preferred for this type of patients.

 In moderate to severe ischaemic mitral regurgitation, there is still no optimal treatment choice in order to decide for a mitral valve intervention or which concomitant mitral procedure to be performed. The repair method that will be performed to the valve in ischaemic MR may vary in accordance with the experience of the surgeons and the valve lesions. But our study has demonstrated that, when the only repair-performed patients and the ring-performed patients are compared, PAP and the recurrent MR rates are statistically higher in only repair-performed group in mid-term. Therefore, we believe that, whether with the valve repair or not, the stabilization of the mitral annulus by a ring annuloplasty will decrease the frequency of the recurrent MR and increase the functional capacity in patients with moderate to severe ischeamic MR.
